# Update on Immunohistochemistry for the Diagnosis of Lung Cancer

**DOI:** 10.3390/cancers10030072

**Published:** 2018-03-14

**Authors:** Kentaro Inamura

**Affiliations:** Division of Pathology, The Cancer Institute, Japanese Foundation for Cancer Research, 3-8-31 Ariake, Koto-ku, Tokyo 135-8550, Japan; kentaro.inamura@jfcr.or.jp; Tel.: +81-3-3570-0111 (ext. 5604); Fax: +81-3-3570-0558

**Keywords:** immune checkpoint, immunostaining, INSM1, morphology, non-small cell lung carcinoma (NSCLC), pathology, thoracic tumor, small cell lung carcinoma (SCLC), tyrosine kinase, WHO classification

## Abstract

Immunohistochemistry is a widely available technique that is less challenging and can provide clinically meaningful results quickly and cost-efficiently in comparison with other techniques. In addition, immunohistochemistry allows for the evaluation of cellular localization of proteins in the context of tumor structure. In an era of precision medicine, pathologists are required to classify lung cancer into specific subtypes and assess biomarkers relevant to molecular-targeted therapies. This review summarizes the hot topics of immunohistochemistry in lung cancer, including (i) adenocarcinoma vs squamous cell carcinoma; (ii) neuroendocrine markers; (iii) ALK, ROS1, and EGFR; (iv) PD-L1 (CD274); (v) lung carcinoma vs malignant mesothelioma; and (vi) NUT carcinoma. Major pitfalls in evaluating immunohistochemical results are also described.

## 1. Introduction

In an era of precision medicine, immunohistochemistry plays a critical role in the classification of tumors into subtypes and for assessing biomarkers for timely and accurate therapeutic decision-making [1,2,3,4,5]. Compared with other techniques, immunohistochemistry has a number of advantages, including being widely available, technically less challenging, and cost-efficient with a rapid turn-around time. Thus, molecular-specific immunohistochemical assays have huge potential as practical screening tools for the detection of druggable genetic alterations and for the assessment of biomarkers for molecular-targeted therapy. In addition, immunohistochemistry can be interpreted using fewer tumor cells than are required for other molecular techniques. Moreover, immunohistochemistry allows for the evaluation of cellular localization and staining patterns in the context of tumor structures; thus, a greater range of information is provided.

Lung cancer is the leading cause of cancer-related deaths worldwide, regardless of gender. It is categorized into two main groups: small cell lung carcinoma (SCLC, 15% of all lung cancers) and non-SCLC (NSCLC, 85% of all lung cancers). Accumulating evidence suggests that lung cancer represents a group of histologically and molecularly heterogeneous diseases [6,7,8,9,10,11,12,13,14,15,16,17]. In addition, increasing knowledge of the molecular pathology of lung cancers has led to their classification into specific subtypes according to appropriate treatments and molecular-targeted therapies. This review provides updated knowledge of the use of immunohistochemistry in lung cancer. Hot topics of immunohistochemistry in lung cancer are discussed, including (i) the differential diagnosis between adenocarcinoma and squamous cell carcinoma (SqCC); (ii) neuroendocrine markers; (iii) driver genetic alterations (ALK, ROS1, and EGFR); (iv) PD-L1 (CD274) expression; (v) the differential diagnosis between lung carcinoma and malignant mesothelioma; and (vi) NUT carcinoma. Major pitfalls in correctly evaluating immunohistochemical results are also described.

## 2. Adenocarcinoma vs. Squamous Cell Carcinoma

The 2015 World Health Organization (WHO) classification was recently modified based on newly identified molecular profiles and druggable genetic alterations in lung cancer [6]. In particular, the 2011 International Association for the Study of Lung Cancer (IASLC), the American Thoracic Society, and the European Respiratory Society classification [18] was mostly adopted in the current WHO classification for lung adenocarcinoma. Advancements in oncology, molecular biology, pathology, radiology, and surgery were considered for the classification of lung cancer into specific subtypes with the aid of immunohistochemistry for therapeutic purposes. In older WHO classifications, the definition of lung cancer subtypes was based on surgical specimens; however, the current WHO categorization is based on small biopsy and cytology specimens, which need to be diagnosed with the help of immunohistochemistry as most lung cancers are detected at later stages.

When possible, differential diagnosis between adenocarcinoma and SqCC is beneficial because targetable driver genetic alterations are mostly identified in adenocarcinoma, and inappropriate drugs need to be avoided for patients with SqCC. Before the 2015 WHO classification, the definitions of adenocarcinoma and SqCC were based on their morphological features with or without mucin staining. Adenocarcinoma was defined as carcinoma with an acinar/tubular structure or mucin production, whereas SqCC was defined as carcinoma with keratinization or intercellular bridges. In the current classification, a solid carcinoma without glandular structures or mucin production, but with immunohistochemical positivity for “adenocarcinoma markers”, i.e., TTF-1 (NKX2-1) and/or Napsin A, is diagnosed as an adenocarcinoma. Similarly, a solid carcinoma without keratinization or intercellular bridges, but with immunohistochemical positivity for “SqCC markers”, such as p40, CK5/6, and TP63 (p63), is diagnosed as SqCC. These modifications using immunohistochemical evaluations have markedly minimized the proportion of NSCLC diagnosed as large cell carcinoma [19].

There exist several pitfalls in differential diagnosis between adenocarcinoma and SqCC [20,21,22]. While performing a differential diagnosis between adenocarcinoma and SqCC using an anti-TTF-1 antibody, a clone of the antibody should be paid attention to. SPT24 and 8G7G3/1 are major clones of the anti-TTF-1 antibody. Whereas TTF-1 (clone SPT24) is less specific (positive in 17% of SqCC) but more sensitive (positive in 72‒84%), TTF-1 (clone 8G7G3/1) is more specific (positive in 1% of SqCC) but less sensitive (positive in 65‒77%) for adenocarcinoma when differentiated from SqCC [21,23,24,25]. Among “SqCC markers”, p40 is the best marker in terms of specificity (positive in 3% of adenocarcinoma) and sensitivity (positive in 100%) [26]. Conversely, TP63 is sensitive (positive in 100%) but less specific (positive in 31% of adenocarcinoma) [26]. Collectively, it should be noted that a significant number of SqCCs or adenocarcinomas show a positivity for TTF-1 (clone SPT24) or TP63, respectively. As another pitfall, trapped benign pneumocytes (positive for TTF-1 and Napsin A) and tumor-infiltrated macrophages (positive for Napsin A) should not be misinterpreted.

## 3. Neuroendocrine Markers

In the 2015 WHO classification [6], the category of “neuroendocrine tumors” was newly recognized. Invasive neuroendocrine tumors comprise three subtypes: SCLC, large cell neuroendocrine carcinoma (LCNEC), and carcinoid tumor (typical/atypical). Although high-grade neuroendocrine tumors (HGNETs), comprising SCLCs and LCNECs, belong to the same category as carcinoid tumors, their clinical characteristics are substantially different. HGNET is an aggressive and deadly subtype characterized by patients with a history of heavy smoking. In contrast, carcinoid tumors usually follow a benign clinical course and frequently occur in patients without a history of smoking. Despite their different clinical characteristics, these tumors share the features of neuroendocrine differentiation. As the definition of LCNEC in the WHO classification, the diagnosis of LCNEC requires not only neuroendocrine morphology but also immunohistochemical expression of at least one of the three neuroendocrine markers, i.e., CHGA (chromogranin A), SYP (synaptophysin), or NCAM1 (CD56).

**Figure 1:**
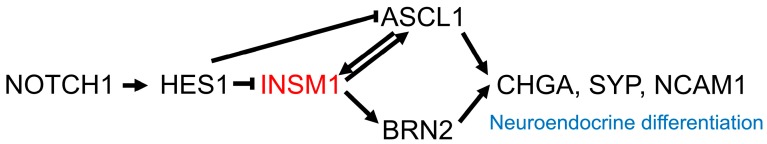
Schematic of the complex pathways of neuroendocrine differentiation (blue) in lung tumors. INSM1 (red) is inactivated by HES1 and promotes the expression of the three neuroendocrine molecules (CHGA, SYP, and NCAM1) via activation of the transcription factors, ASCL1, and BRN2. INSM1 and ASCL1 activate each other.

Neuroendocrine differentiation of lung tumors is orchestrated by complex pathways as concisely displayed in Figure 1 [27,28]. The NOTCH1-HES1 signaling pathway represses neuroendocrine differentiation by inactivating INSM1 and ASCL1. INSM1 is a zinc-finger transcriptional factor originally isolated from pancreatic insulinomas [29]. NOTCH1 activates HES1, which inactivates INSM1 and ASCL1. INSM1 promotes the expression of the three neuroendocrine molecules (CHGA, SYP, and NCAM1) via the activation of the transcription factors ASCL1 and BRN2. HES1 is a known transcriptional repressor of ASCL1.

**Figure 2:**
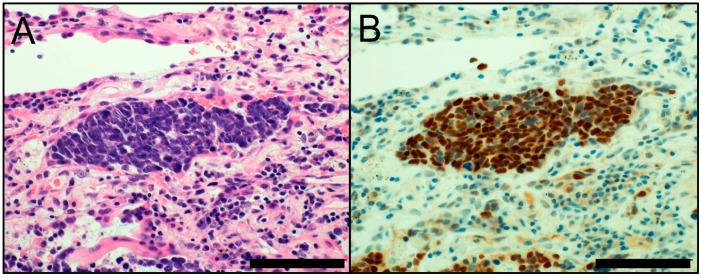
(**A**) Morphology of small cell lung carcinoma (hematoxylin and eosin staining); (**B**) INSM1 immunostaining (nuclear; positive). Scale bar = 100 µm.

A recent study demonstrated that INSM1 was positive in 94.9% of SCLCs and 91.3% of LCNECs, compared with 74.4% and 78.3% with the combined panel of the three neuroendocrine markers (CHGA, SYP, and NCAM1). Thus, INSM1 was suggested to be more useful than the individual or combined use of CHGA, SYP, and NCAM1 for the diagnosis of lung HGNETs [30]. INSM1 appears to be a novel, sensitive, and specific immunohistochemical marker that may serve as a standalone first-line marker of neuroendocrine differentiation (Figure 2).

## 4. ALK, ROS1, and EGFR

Various somatic genetic alterations in tyrosine kinase have emerged as druggable molecular targets by tyrosine kinase inhibitors (TKIs), particularly in lung adenocarcinoma [31]. With a prudent evaluation of genetic-alteration-specific immunostaining, immunohistochemistry has enormous potential to be used as a practical screening tool to detect certain actionable genetic alterations amenable to molecular-targeted therapies.

*ALK*-rearranged lung adenocarcinoma, comprising 4‒5% of lung adenocarcinomas, is clinicopathologically characterized by a TTF-1 cell lineage, an acinar structure with mucin/signet-ring cell morphology, non-/light-smoking history, and young onset [32,33,34,35]. Various clinical trials have demonstrated the clinical efficacy of TKIs or ALK inhibitors in patients with *ALK*-rearranged NSCLC [36,37,38]. Immunohistochemistry for ALK represents a cost-effective and widely available method that is an effective screening tool to detect the presence of *ALK* rearrangement, in addition to conventional fluorescence in situ hybridization (FISH) [39]. According to recent studies, ALK antibody clones D5F3 and 5A4 show the highest sensitivity and specificity compared with several available anti-ALK antibodies [40]. Recently, the U.S. Food and Drug Administration (FDA) approved an immunohistochemical assay using the ALK D5F3 antibody as a companion diagnostic assay for patients with *ALK*-rearranged NSCLC. Therefore, patients with ALK-positive NSCLC, as confirmed by immunostaining using clone D5F3, are candidates for ALK inhibitor treatment. Of note, immunohistochemistry using the ALK D5F3 antibody appears to be not only a candidate but also the most important test for ALK testing. There are several studies suggesting the superiority of the ALK immunohistochemistry with the D5F3 antibody compared with *ALK* FISH at predicting response to ALK inhibitors [41,42,43,44].

**Figure 3:**
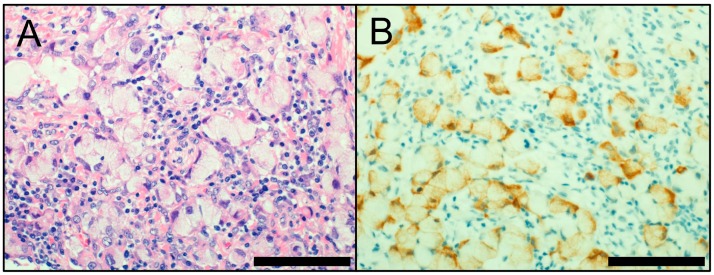
(**A**) *ALK*-rearranged lung adenocarcinoma with a signet-ring cell pattern (hematoxylin and eosin staining); (**B**) ALK immunostaining (cytoplasmic; positive). Scale bar = 100 µm.

Immunohistochemically, *ALK*-rearranged lung cancers show cytoplasmic ALK staining. Intracellular mucin vacuoles are frequently observed in *ALK*-rearranged adenocarcinoma; thus, inadequate cytoplasmic immunostaining can cause these to be carelessly missed (Figure 3). In addition, nonspecific immunostaining can be observed in some neuroendocrine carcinomas without *ALK* rearrangement [2].

*ROS1* rearrangement is an oncogenic driver in a subset (1‒2%) of lung adenocarcinomas [45,46,47]. *ROS1*-rearranged adenocarcinoma is clinicopathologically characterized by solid growth with signet-ring cells or a cribriform morphology with abundant extracellular mucus, and typically occurs in younger non-smoking females [48]. Clinical trials have demonstrated the clinical efficacy of TKIs or ROS1 inhibitors in patients with NSCLC with *ROS1* rearrangement confirmed by FISH assays [47,49]. As with *ALK*-rearranged NSCLCs, immunohistochemistry provides high sensitivity and specificity for the detection of *ROS1* rearrangements. Immunohistochemical assay using the specific rabbit monoclonal antibody clone D4D6 is a cost-efficient and widely available method for screening patients with *ROS1*-rearranged NSCLCs [50,51]. However, there is no benign tissue that can be used as a positive control for ROS1. This contrasts with ALK, where ganglion cells and nerves of the appendix can be used as a positive control. Therefore, tumors or cell lines with confirmed *ROS1* rearrangement need to be used as an external positive control [52]. Further, the ROS1 staining pattern depends on the partner genes of *ROS1* fusion. Adenocarcinomas with *CD74-ROS1* fusion, which is the most frequent fusion gene, usually shows globular cytoplasmic ROS1 immunoreactivity, whereas adenocarcinomas with *EZR-ROS1* fusion usually show membranous immunostaining [48]. Similar to *ALK*-rearranged adenocarcinomas, an intracellular mucin vacuole is frequently observed in *ROS1*-rearranged adenocarcinomas; therefore, inadequate immunostaining should not be missed. It should be also noted that benign hyperplastic pneumocytes and macrophages frequently show weak ROS1 immunostaining [53]. Furthermore, the possibility of false-positive tumors in ever-smoking patients has been suggested [54]. Currently, screening by immunohistochemistry for ROS1, followed by the subsequent confirmation of ROS1-positive cases by FISH, is required. TKIs or ROS1 inhibitors should be applied only to cases that are positive for *ROS1* rearrangement, as confirmed by both immunohistochemical and FISH analyses [2].

The *EGFR* mutation is one of the most common driver mutations in lung adenocarcinoma, and *EGFR*-mutated adenocarcinoma is characterized by East Asian ethnicity, female gender, and non-/light-smoking history [55]. Pathologically, *EGFR*-mutated lung adenocarcinoma typically shows nuclear TTF-1 immunoreactivity and hobnail cell morphology. In addition, adenocarcinoma with micropapillary morphology has a higher frequency of *EGFR* mutations than adenocarcinoma without this morphology [56]. In the gene coding for the receptor, *EGFR* mutations are divided into four major types: point mutations in exon 18, deletions in exon 19, insertions in exon 20, and point mutations in exon 21. Approximately 90% of *EGFR* mutations in NSCLCs involve in-frame deletions in exon 19 and the point mutation L858R in exon 21. These mutations, particularly exon 19 deletions, are associated with a superior and prolonged clinical response to EGFR TKIs [57,58]. *EGFR* mutation-specific antibodies, recognizing a 15-bp deletion in exon 19 (clone: 6B6) and an L858R point mutation in exon 21 (clone: 43B2), have been developed [59]. However, immunohistochemical analysis using these antibodies has not been recommended for screening *EGFR* mutations due to its low sensitivity.

## 5. PD-L1 (CD274)

PD-L1 (CD274) is an immune modulator that promotes immunosuppression by binding to PD-1 (PDCD1). PD-L1 on the surface of tumor cells inhibits an immune-mediated attack by binding to PD-1 on cytotoxic T-cells [60,61]. Although various studies have reported the association of PD-L1 positivity in tumor cells with prognosis in lung cancer, the results are conflicting and inconclusive [62,63,64,65,66,67,68,69,70]. A possible reason for the discordant results lies in cohort-dependent non-standardized immunohistochemical assays. Another possible reason is that the association of PD-L1 positivity with clinical outcome truly differs depending on the cohorts. Anti-PD-1/PD-L1 antibodies inhibit PD-L1 binding to PD-1, thus allowing immune-mediated attacks against tumor cells at this immune checkpoint. Multiple clinical trials using these antibodies for the treatment of malignancies, including NSCLCs, have shown great promise in prolonging survival [71,72,73]. According to a clinical trial for PD-1 inhibitor, pembrolizumab, for the treatment of NSCLCs [74], NSCLCs with at least 50% positivity for PD-L1 were associated with a higher response rate and longer survival than NSCLCs with less than 50% positivity. Of importance, although a response rate is lower than NSCLCs with at least 50% positivity for PD-L1, a certain subset of NSCLCs with less than 1% positivity still responded to pembrolizumab. Given this result, there remains an urgent need for the identification of more reliable biomarkers that predict the responsiveness to immune checkpoint inhibitors.

Specific immunohistochemical assays for different PD-1/PD-L1 inhibitors have been designed to estimate sensitivities to these treatments [75]. Currently, there are five different PD-1/PD-L1 inhibitors that require specific immunohistochemical assays using different anti-PD-L1 antibodies. These include nivolumab with clone 28-8, pembrolizumab with clone 22C3, atezolizumab with clone SP142, durvalumab with clone SP263, and avelumab with clone 73-10 [60,76,77,78]. For assays using the 22C3, 28-8, SP263, and 73-10, complete circumferential or partial membranous immunostaining of any intensity is considered to be positive. In an assay using the SP142, the presence of PD-L1-positive immune cells is also considered while determining the PD-L1 positivity. The U.S. FDA has currently approved a companion diagnostic PD-L1 test for pembrolizumab (assay using the 22C3 antibody) and the complementary diagnostic PD-L1 tests for nivolumab (assay using the 28-8 antibody) and atezolizumab (assay using the SP142 antibody), whereas clinical trials with the two agents durvalumab (assay using the SP263 antibody) and avelumab (assay using the 73-10 antibody) have also demonstrated promising results [3,79,80,81]. The requirement for different kits, instruments, and interpretative criteria for each drug is challenging for pathology laboratories and pathologists. To know whether one of these assays can be used to select eligible patients for anti-PD-1/PD-L1 inhibitors, comparisons of the difference among these assays have been made by several studies [82,83,84,85,86] including the Blueprint project [82], which is an industrial–academic collaborative partnership among the IASLC and the American Association for Cancer Research, pharmaceutical companies, and diagnostics venders. According to these studies, the 22C3, 28-8, and SP263 assays showed a similar membranous staining on tumor cells; however, the SP142 assay consistently had fewer PD-L1 tumor cells expressing PD-L1. As for the PD-L1 expression on immune cells, low concordance rates were observed among these assays, indicating a requirement for specific standardization of immune cell scoring.

As an external positive control for PD-L1, human tonsils, placenta, or PD-L1-positive cell lines can be used. In addition, at least 100 viable tumor cells need to present in one PD-L1-immunostained slide to determine the percentage of PD-L1-positive cells. Of note, the careful observation of both hematoxylin-eosin (HE)-stained slides and immunostained slides is required to correctly evaluate PD-L1 positivity, as PD-L1 can be immunostained in inflammatory cells, including macrophages and lymphocytes. When PD-L1-positive macrophages and/or lymphocytes exist around PD-L1-negative tumor cells, misinterpretation should be carefully avoided. In addition, tumor cells with cytoplasmic granular immunostaining, but without membranous staining, should not be misinterpreted as positive [2,3].

## 6. Lung Carcinoma vs. Malignant Mesothelioma

Malignant mesothelioma (MM) is a rare and fatal malignant tumor arising from mesothelial cells. Malignant mesothelioma is one of the important tumors that need to be distinguished from lung cancer while diagnosing lung cancer. Asbestos exposure is the main risk factor for developing MMs, which are generally classified into three major histologic subtypes: epithelioid (60–80%), sarcomatoid (<10%), and biphasic (10–15%). Epithelioid MM is the most common subtype and shows a relatively better prognosis than sarcomatoid or biphasic MM [6]. MM needs to be diagnosed based on the patient’s clinical and radiologic findings in conjunction with morphological and immunohistochemical features. The differential diagnoses between MM and benign mesothelial proliferation, as well as between MM and lung carcinoma, are sometimes challenging.

Reactive mesothelial proliferations occasionally mimic MMs, because reactive mesothelial proliferations sometimes exhibit cellular and structural atypia typically observed in MMs. Therefore, differential diagnosis between MM and reactive mesothelial proliferation is often difficult. The loss of BAP1, confirmed by immunohistochemistry, and homozygous *CDKN2A* (*p16*) deletions, identified by FISH, have recently emerged as potential indicators of MM. Loss of nuclear BAP1 immunostaining is often observed in MMs, particularly the epithelioid/biphasic subtype, but not in active mesothelial proliferations. However, BAP1 loss is relatively uncommon in the sarcomatoid subtype. Therefore, BAP1 immunohistochemistry has a relatively high specificity, but low sensitivity [87,88,89]. In contrast to BAP1 loss, the *CDKN2A* deletion is observed more frequently in the sarcomatoid subtype than in the epithelioid/biphasic subtype [88,89,90].

Lung carcinomas can also show pseudomesotheliomatous spreads. As no immunohistochemical marker is completely specific for each type of tumor, the International Mesothelioma Interest Group recommends at least two mesothelial and two carcinoma markers, in addition to cytokeratins, to be included in the differential diagnosis between MM and carcinoma. Mesothelial markers include calretinin (nuclear and cytoplasmic staining), podoplanin (clone D2-40; membranous staining), CK5 or CK5/6 (cytoplasmic staining), and WT1 (nuclear staining) [91]. For differential diagnosis between MM and lung adenocarcinoma, TTF-1, Napsin A, CEA, claudin 4 (CLDN4), Ber-EP4, and MOC31 are useful markers suggesting lung adenocarcinoma. A recent study has demonstrated that the positive rates of DAB2 and Intelectin-1 (INLT1) expression were 80% and 76% in epithelioid MM, and 3% and 0% in lung adenocarcinoma, respectively. This study indicates that DAB2 and Intelectin-1 are novel positive immunohistochemical markers of epithelioid MM, and should allow for its differentiation from lung adenocarcinoma [92]. For sarcomatoid MM, a recent study has suggested MUC4 as a novel negative immunohistochemical marker of sarcomatoid MM for its differentiation from lung sarcomatoid carcinoma [93].

Collectively, immunohistochemistry plays an essential role in the differential diagnosis of MM; thus, the appropriate selection of immunohistochemical markers is essential. Morphological, clinical, and radiological features are also needed for correct diagnosis.

## 7. NUT Carcinoma

NUT carcinoma has been recently recognized as a subtype of lung cancer in the 2015 WHO classification [6]. Because NUT carcinoma is frequently misdiagnosed as different malignancy, it needs to be considered in the differential diagnosis of malignancies in lung. NUT carcinoma is defined by a gene rearrangement between the *NUT* (*NUTM1*) gene on chromosome 15q14 and one of the other partner genes. The *NUT* gene is fused to the bromodomain family member *BRD3* on chromosome 19p13.1 (comprising 70% of NUT carcinomas), *BRD4* on chromosome 9q (6%), or the other partner genes. The methyltransferase *NSD3* on chromosome 8q11.23 was newly recognized as a fusion partner of *NUT* [6,94,95,96,97,98,99]. These rearrangements lead to global epigenetic reprogramming and loss of cell differentiation [98,99,100]. Although NUT carcinoma occasionally exhibits abrupt foci of keratinization in morphology, NUT carcinomas show few cytogenetic alterations with the exception of chromosomal translocation involving *NUT*. This contrasts with lung SqCCs, which are characterized by complex and multiple cytogenetic alterations [6,100]. In terms of the simple cytogenetic alterations, NUT carcinomas closely resemble hematological tumors, which are similarly characterized by simple cytogenetic alterations.

Clinically, NUT carcinoma shows an extremely aggressive behavior with dismal prognosis, and a median overall survival of 2.2 months. NUT carcinoma occurs with no predominance of gender, and affects people of any age and smoking history, although it was originally reported in young individuals [6,94,101]. Although NUT carcinoma lacks benefit from chemotherapy or radiotherapy [94,98], molecular-targeted therapies against bromodomain may be beneficial as either a single agent or in combination with other agents [102,103].

**Figure 4:**
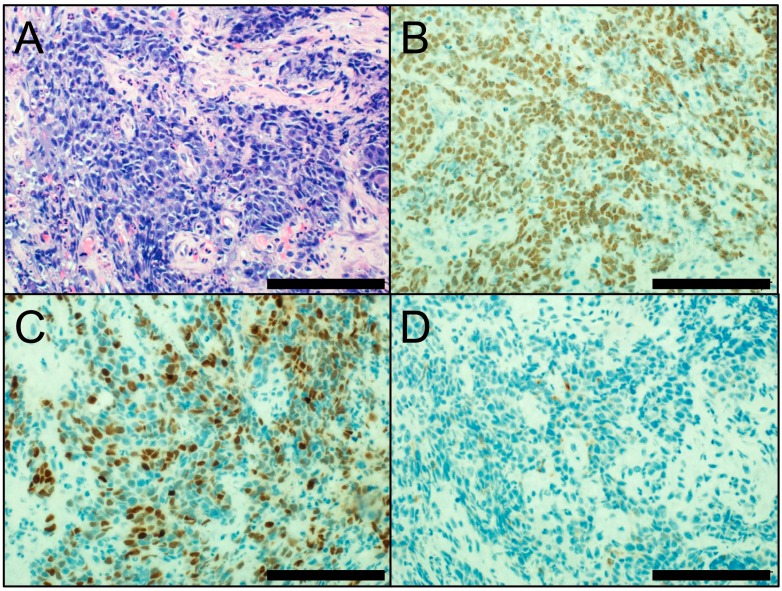
(**A**) Morphology of NUT carcinoma (hematoxylin and eosin staining). Immunohistochemistry of NUT carcinoma; (**B**) NUT staining (nuclear; positive); (**C**) p40 staining (nuclear; focally positive); and (**D**) SYP staining (cytoplasmic; very focally positive). Scale bar = 100 µm.

Morphologically, NUT carcinoma is characterized by sheets and nests of small- to intermediate-sized monotonous, primitive-appearing tumor cells (Figure 4A), with the occasional abrupt foci of keratinization. The lack of a pathognomonic appearance leads to the frequent misdiagnosis of NUT carcinoma as basaloid SqCC, SCLC, lymphoma, or germ cell tumor [6,94,101].

Immunohistochemical detection of the nuclear NUT protein facilitates a rapid and cost-effective diagnosis of NUT carcinoma, with the aid of highly specific and sensitive monoclonal NUT antibodies [104]. NUT carcinoma typically shows speckled nuclear positivity for NUT (Figure 4B). Both morphological and immunohistochemical features contribute to the correct diagnosis of NUT carcinoma. Because seminomas may show weak and focal NUT immunostaining, careful observation of the NUT staining pattern is required [105]. In addition, NUT carcinoma is usually positive for broad-spectrum cytokeratins and often shows nuclear staining for p40, suggesting lineage from SqCC (Figure 4C) [106]. Due to its morphological and immunohistochemical similarities with basaloid SqCC, it is important that NUT carcinoma not be diagnosed as basaloid SqCC. NUT carcinoma can be immunostained for the neuroendocrine markers CHGA and SYP (Figure 4D). In addition, because NUT carcinoma shares morphological similarity (small- to intermediate-sized monotonous appearances and crush artifacts) and neuroendocrine differentiation with SCLC, a misdiagnosis as SCLC should be avoided. Immunohistochemical assessment of NUT nuclear expression needs to be considered in cases of poorly differentiated carcinomas, particularly in young patients without a history of smoking [6,94,101].

## 8. Conclusions and Future Directions

Immunohistochemical techniques play critical roles as diagnostic and screening tools for lung cancer. However, the limitations of immunohistochemistry should be fully apprehended to avoid inappropriate results from immunohistochemical assays [2,107]. The results of immunohistochemical assays are affected by variable pre-analytical handling of the specimen, including delay in fixation, inappropriate fixation time, inappropriate fixative solution, and issues regarding paraffin embedding [108]. The immunohistochemical results are also influenced by analytic variables, including antigen retrieval, concentration of the antibody, incubation time/temperature, and signal enhancement. There also exists a problem of inter- and intra-observer variability in evaluating immunostainings. Although digital pathology has a potential to overcome subjectivity and improve reproducibility, the capability of digital pathology remains impractical in most cases. The immunostaining data still require interpretation by experienced pathologists, who need to acquire proficiency in immunohistochemistry updates. Also, immunohistochemical evaluation should be performed concurrently with detailed observations of corresponding HE slides. Conversely, immunohistochemistry has several advantages in comparison with other assays, including as a cost-effective and widely available technique with a rapid turn-around time. Immunohistochemical assays can also be performed with fewer tumor cells, and they allow for the evaluation of cellular localization of proteins in the context of tumor structure. In the current WHO classification, immunohistochemical analysis is indispensable to the determination of lung cancer subtypes. Furthermore, immunohistochemical assays have been approved by the U.S. FDA as companion or complimentary diagnostic assays for molecular-targeted therapies. Moreover, an increasing number of targeted therapies will require immunohistochemical evaluation in order to determine the eligibility of patients for certain treatments. Thus, molecular-specific immunohistochemical assays will be performed more frequently to determine specific subtypes, make differential diagnoses, and evaluate relevant biomarkers in lung cancer. Collectively, as part of the current era of precision medicine, immunohistochemical techniques have great promise for improving the diagnosis and treatment of lung cancer.

## Abbreviations

FDA	Food and Drug Administration
FISH	fluorescence in situ hybridization
HE	hematoxylin-eosin
HGNET	high-grade neuroendocrine tumor
IASLC	International Association for the Study of Lung Cancer
INSM1	insulinoma-associated protein 1
LCNEC	large cell neuroendocrine carcinoma
MM	malignant mesothelioma
NSCLC	non-small cell lung carcinoma
SCLC	small cell lung carcinoma
SqCC	squamous cell carcinoma
TKI	tyrosine kinase inhibitor
WHO	World Health Organization
